# Non-psychiatric Referral among First Encounter Patients Attending the Psychiatry Out Patient Services of a Tertiary Care Hospital: A Descriptive Cross-sectional Study

**DOI:** 10.31729/jnma.6244

**Published:** 2021-10-31

**Authors:** Grishma Pokharel, Madhur Basnet, Sagar Poudel, Naresh Kafle, Rahul Kumar Jaiswal, Sunil Bogati, Indira Ojha, Dipesh Soni

**Affiliations:** 1 Melamchi Primary Health Care Center, Melamchi, Sindupalchok, Nepal; 2 Department of Psychiatry, BP Koirala Institute of Health Sciences, Dharan, Nepal; 3 School of Public Health and Community Medicine, All India Institute of Medical Sciences, New Delhi, India; 4 Waling Primary Health Care Center, Waling, Syangja, Nepal; 5 Department of Surgery, All India Institute of Medical Sciences, Rishikesh, India; 6 Kolhabi Primary Health Care Clinic, Kolhabi, Bara, Nepal; 7 Bulingtar Primary Health Care Center, Bulingtar, Nawalparasi, Nepal; 8 Department of Internal Medicine, Postgraduate Institute of Medical Education and Research, Chandigarh, India

**Keywords:** *faith healing*, *psychiatry*, *referral*

## Abstract

**Introduction:**

Pathway of psychiatric care is defined as the sequence of contacts with individuals and organizations initiated by the distressed person's efforts and his significant others to seek appropriate health care. This study aimed to find the prevalence of non-psychiatric referral as first encounter among patients attending the psychiatry outpatient department of a tertiary care hospital.

**Methods:**

A descriptive cross-sectional study was carried out from 29^th^ March 2015 to 25^th^ April 2015 in the outpatient department of the department of psychiatry of a tertiary via direct interview using the World Health Organization's encounter form. Ethical approval was taken from undergraduate medical research protocol review board (Reference number 105/071/072). Psychiatric diagnoses were made by respective consultants using the International Classification of Diseases-10 Clinical Descriptions and Diagnostic Guidelines criteria. Data was entered in the Microsoft Excel 2007 and analyzed by Stata version 15. Point estimate at 95% Confidence Interval was calculated along with frequency and percentage for binary data.

**Results:**

Out of 50 patients, 26 (52%) (38.2-65.8 at 95% Confidence Interval) of new cases in the outpatient department had non-psychiatric referrals. Among them, 13 (26%) referred from faith healers, 7 (14%) from the general hospital and 6 (12%) from medical out patient department.

**Conclusions:**

The prevalence of non-psychiatric referral for the patients seen for the first time in the psychiatry outpatient department was similar to findings from studies done in different parts of South East Asia.

## INTRODUCTION

Mental illnesses are commonly linked with a higher disability and burden of disease than many physical illnesses.^1^ Pathway to care in psychiatry is defined as the sequence of contacts with individuals and organizations, initiated by the distressed person's efforts and those of his significant others to seek appropriate help.^2^

The World Health Organization (WHO) noted that one in every four people are affected by a mental disorder at some stage of life. So, timely identification and treatment of mental illnesses is of vital importance. Treatment from unqualified medical practitioners and faith healers is a common practice, and is attributable to the delay in proper treatment.^3^ Study of pathway to care helps us identify the points of contact that the patient comes in an attempt to seek care and thus find the possible hurdles in the undue delay to proper treatment.

The aim of this study was to find out the prevalence of non-psychiatric referrals among new patients visiting the psychiatry out-patient department (OPD) of a tertiary care hospital.

## METHODS

A descriptive cross-sectional study was carried out from 29^th^ March 2015 to 25^th^ April 2015 in OPD in the department of Psychiatry BP Koirala Institute of Health Sciences (BPKIHS), Dharan, Nepal. Ethical approval was taken from undergraduate medical research protocol review board (UM-RPRB) BPKIHS formed under the institutional review committee (IRC) (Ref. No.105/071/072). Patients visiting the psychiatry outpatient department for the first time were included in the study. Patient on follow up visit and those refusing to give the consent were excluded from the study. Convenience sampling was done and the sample size was calculated as,

n = Z^2^ × p × q / e^2^

  = (1.96)^2^ × (0.87) × (1-0.87) / (0.1)^2^

  = 43.44

  = 45

Where,

n = minimum required sample size,
Z = 1.96 at 95% Confidence Interval (CI),
p = past prevalence of non-psychiatric referrals among new cases taken from a previous study, 87%^4^
q = 1 - p
e = margin of error, 10%

Taking a 10% non-response rate, the calculated sample size was 50. Therefore, we took 50 participants in the study. Data was collected in the OPD by face-to-face interview by the researchers with the patients or patient's informant using standardized encounter form by World Health Organization. The participants were informed regarding the study prior to the interview and taken an informed consent. The Psychiatric diagnoses were made by the respective psychiatric residents and confirmed by the respective consultants using ICD-10, Clinical Description and Diagnostic Guidelines (CDDG) criteria. All first point of care apart from the direct psychiatric consultation were taken as non psychiatric referral.

Data was be entered in Microsoft excel 2007 and analyzed by STATA 15. Point estimate at 95% Confidence Interval was calculated along with frequency and percentage for binary data.

## RESULTS

The prevalence of non-psychiatric referrals among firstencounter patients visiting the psychiatry OPD was 26 (52%) (38.2-65.8 at 95% CI). Out of them, native or religious healers accounted for 13 (26%) while 7 (14%) first consulted general hospital and 6 (12%) to other medical practitioners.

The most common symptoms first experienced by the subjects were depressive symptoms 4 (8%), panic 4 (8%), mania 4 (8%) and anxiety 2 (4%). Five (10%) were involved in impulsive activities and 2 (4%) had visual and auditory hallucinations. Rest 5 (10%) had other symptoms like premature ejaculation, headache, alcohol dependence syndrome and seizures.

**Table 1:** Data showing first symptom developed by the patient consulting the non psychiatric services at their first referral.

First symptom developed by the patient	Native/religious healer n (%)	Medical practitioner n (%)	General hospital n (%)	Total n (%)
Anxiety	2 (15.38)	0 (0.00)	0 (0.00)	2 (04.00)
Depressive symptoms	1 (7.69)	2 (33.33)	1 (14.29)	4 (08.00)
Impulsive act	0 (0.00)	4 (66.67)	1 (14.29)	5 (10.00)
Hallucinations	2 (15.38)	0 (0.00)	0 (0.00)	2 (4.00)
Panic	3 (23.08)	0 (0.00)	1 (14.29)	4 (08.00)
Maniac symptoms	3 (23.08)	0 (0.00)	1 (14.29)	4 (08.00)
Others	2 (15.38)	0 (0.00)	3 (42.86)	5 (10.00)
Total	13 (100.00)	6 (100.00)	7 (100.00)	26 (52.00)

Fourteen (28%) of our subjects were diagnosed to have mild, moderate or severe depression. Next to it, 8 (16%) had bipolar disorder. Panic disorder 6 (12%) and mania 6 (12%) were next common followed by schizophrenia 5 (10%). Other remaining 9 (18%) were diagnosed to have early ejaculation, migraine with insomnia, unresponsive episodes of different durations and mood disorder with psychotic symptoms.

In about 34 (68%) of the cases, it was found that friends, relatives or neighbors initiated the first contact while in the rest 16 (32%) it was patient himself/herself.

We found that 2 (25%) of those patients with anxiety symptoms, 2 (67%) of those with hallucinations, 3 (18%) of patients with mania, 3 (18%) of patients with panic disorder and 4 (40%) of those with depressive symptoms consulted the non psychiatric services.

Similarly, 11 (38%) patients of the patients from within 50km of tertiary care centre, 1 (33%) of those from >50km hill and and 10 (5.5%) people from >50km terai first consulted native religious healers.

Data regarding the distance from tertiary care centre and their place of first referral showed that 18 (63%) of people from within 50km, 1 (34%) people from >50km hill and 7 (39%) people from >50km terai region had their first referral to non psychiatric services.

It was observed that 21 (72%) patients from within 50 km of tertiary care center had their first referral within 6 months of first consultation to any non psychiatric services. Similar was the result for people from >50km terai 13 (72%) while only 2 (66%) of those from >50km hill had their first referral within 6 month of consultation to non psychiatric services.

**Table 2:** Data showing time to first referral from non psychiatric consultations among patients with psychiatric illnesses.

Address	Time to first referral				Total
	<6 months n(%)	6 months-1 year n(%)	>1 year to <5years n(%)	>5 years n(%)	n(%)
<50 km	21 (58.33)	0 (0.00)	4 (50.00)	4 (80.00)	29 (58.00)
>50km Hill	2 (5.56)	1 (100.00)	0 (0.00)	0 (0.00)	3 (6.00)
>50 km Terai	13 (36.11 )	0 (0.00)	4 (50.00)	1 (20.00)	18 (36.00)
Total	36 (100.00)	1 (100.00)	8 (100.00)	5 (100.00)	50 (100.00)

Our study found that 39 (78%) of the patients sought referral from non psychiatric services within 6 months of the development of their first symptoms. 6 (12%) made the initiation within a year while it took more than 5 years for 4 (8%) of them. 1 (2%) sought the contact in the period between 1 year to 5 years.

The results showed that majority of people from <50 km from tertiary care centre had their first mental health consultation within 6 months 24 (82%) while 13 (72%) of those from >50 km terai also sought the care within 6 months. Those from more than 50 km hill had significant time lag in consultation with only 2 (66%) seeking care within 6 months.

## DISCUSSION

A better understanding of the way in which people understand and seek care for mental disorders is important for planning mental health services, for the organization of training and for the organization of referrals to psychiatrists from other sources of health and social care.^5^ The findings of our study show that 52% of our patients had referral from the non psychiatric services.

Many studies from various countries have described the help-seeking behavior of patients with psychiatric disorders.^15^ A large-scale study by the World Health Organization (WHO), one of the largest in this field, investigated referral pathways in 11 countries and demonstrated that the main contact point before attending psychiatric care was a general practitioner (four European centers, Havana, Aden, and Mexico city), a hospital doctor (Nairobi), and a traditional healer (Ujung Pandang).^1^ This is in accordance to our study which also showed that major referral pathways to mental health are non psychiatric services. The role of relatives, friends and neighbors was found to be greater in the initiation of contact than the patient himself/herself. This is consistent with the findings of the study done in Italy done by Valeria Del Vechho et al.^11^

The study was of short duration and sample mostly representative of urban population was the major limitation of our study. As the study was hospital based, convenience sampling was done because of which the study might not be representative to the community. Recall bias might have contributed to some of the limitations.

## CONCLUSIONS

The findings of our study showed that more than half of the cases of mental illness had sought non psychiatric care as their first consultation. This is similar to the studies conducted by World Health Organization and studies in South East Asia Region. Reducing delays in accessing the services and providing early intervention
